# Progress and Advancement of Medical Education in Texas

**Published:** 1914-08

**Authors:** W. L. Crosthwait

**Affiliations:** Secretary Texas State Board of Medical Examiners


### Progress and Advancement of Medical Education in Texas.

W. L. CROSTHWAIT, SECRETARY TEXAS STATE BOARD OF MEDICAL EXAMINERS.

Notwithstanding the fact that prior to 1876 four separate and distinct regular constitutional conventions assembled in Texas, each drafting a Constitution touching upon the various issues and questions involving the welfare of the people of Texas, not one word of mention or reference do we find to the practice of medicine and surgery in Texas.

No legislation was enacted regulating the practice of medicine until the Act of May 16, 1873, in which act was provided that no person shall be permitted to practice medicine in any of its branches or departments in this State as a means of livelihood without first having attended a regular course of study and lectures at some regularly established and well accredited medical college and received the degree of doctor of medicine, or without having a certificate of qualification from some authorized board of medical examiners. Said act further provided that every person theretofore engaged in the practice of medicine should, within twenty days after the organization of the Board of Medical Examiners, which Board was therein provided for, furnish to the clerk of the district court of the county his diploma or certificate of qualification, and every person who might thereafter engage in the practice of medicine should, within twenty days after entering such practice, furnish to the clerk of the district court his diploma or certificate of qualification. It was provided that the clerk should enter the name of such person in a well bound book to be kept in his office. Such Boards of Medical Examiners were appointed by the county courts.

The Constitution ratified February, 1876, contained the following language (see Section 31):

"The Legislature may pass laws prescribing the qualifications of practitioners of medicine in this State, and to punish persons for malpractice, but no preference shall ever be given by law to any school of medicine."

The Legislature in session at that time enacted the law known as the Act of 1876, regulating the practice of medicine in Texas. This law created district boards to be appointed by the various district judges, and the qualifications to practice medicine were practically as prescribed in the Acts of 1873, with the further provision that applicants should be examined in certain fundamental branches of medicine, whether they were graduates of schools or not. While this act gave to the various district boards a right to ascertain by examination whether or not an applicant for license had sufficient knowledge of the fundamental branches to qualify him to practice medicine and surgery, yet it did not give them the right to establish standards for medical colleges or to prescribe preliminary educational requirements and qualifications for those beginning the study of medicine. This law remained unchanged until 1901, when by an Act of the Twenty-seventh Legislature, ratified February 22, 1901, a law was created establishing three separate State Boards of Medical Examiners, known, respectively, as the Regular Board of Medical Examiners of Texas, the Eclectic Board of Medical Examiners, and the Homoeopathic Board of Medical Examiners. While the powers and functions of these three different Boards were practically the same, they exercised the right to conduct examinations upon the fundamental branches of medicine and surgery, yet they were still without power to fix a minimum standard of medical education to apply to medical colleges.

The present medical act, known as the One Board Medical Act, established by the Thirtieth Legislature, and becoming effective July 13, 1907, created five different classes of practitioners in reference to their legalization:

"1. Those who began the practice of medicine after the act became effective.

"2. Those who were practicing medicine in Texas prior to January 1, 1885.

"3. Those who began the practice of medicine in this State after January 1, 1885, and who have complied with the laws of this State regulating the practice of medicine enforced prior to the passage of the Act of 1901.

"4. Those who were practicing by virtue of having had a diploma from a medical college recorded between the first day of January, 1891, and the first day of January, 1901.

"5. Those who were legalized by examination by one of the three Boards created by the Act of 1901."

Section 7 of the medical law enacted in 1907 contains the following language:

"Applicants to be eligible for examination must present satisfactory evidence to the Board that they are more than 21 years of age, of good moral character and graduates of bona fide reputable schools. Such school shall be considered reputable within the meaning of this act, whose entrance requirements and courses of instruction are as high as those adopted by the better class of medical schools of the United States, whose course of instruction shall embrace not less than four terms of five months each."

Upon this authority the Board, by provision of said act, proceeded to enforce requirements of Texas medical colleges equivalent to those adopted by the better class of medical schools of the country.

At the first meeting of the Board a College Committee was appointed, consisting of one representative of each of the different systems of medicine represented on the Board. This committee found itself confronted with a Herculean task at that time. Very few of the schools of the United States were enforcing any entrance requirements or preliminary education requirements at all. Very few of them were adhering strictly to their published curriculum and course of instruction. The College Committee recommended that a preliminary educational requirement be established commencing with 8 Carnegie units or standard high school units to apply to the matriculants of 1908, increasing 2 units each year, or 10 units to apply to matriculants of 1909, 12 units to those of 1910, and 14 units to apply to matriculants of 1911. The entrance requirements of this Board remained at 14 units until the June meeting of the Board this year (1914), when the requirements were made 14 standard or affiliated high school units, plus one college year with certain provisions applying to conditions of one or two college units, which must be removed before the beginning of the junior year.

When this Board was organized, two medical colleges were in existence in Texas, which were practically nothing more than diploma mills, allowing students to matriculate and graduate with only a few weeks actual attendance. These were promptly closed up.

Next came the problem of dealing with medical schools directly with reference to the question as to whether or not they were conducting colleges equal in every respect to the better class of medical colleges of the country. The Council of Medical Education of the A. M. A. began in 1908 to inspect and classify medical colleges. Their first published report classified all medical colleges in three classes—classes A, B and C. Later reports classified four classes, namely, A plus, A, B and C. A plus schools included all medical colleges which were perfect in every condition. Class A included those schools which were practically acceptable but which needed a few minor changes. Class B included those schools which were in condition to make acceptable by complying with various conditions, etc. Class C included colleges which stood in need of a complete reorganization to put them in position to become acceptable medical colleges.

The College Committee of the State Board of Medical Examiners twice a year visited the different medical schools in Texas, carefully checking up the equipments of said schools, studiously entering into their methods of teaching, the hours filled by the professors, etc. Incidentally, other colleges in adjoining States were visited by various members of the Board of College Committee for the purpose of comparison.

At the meeting of the Board in June, 1913, a resolution was passed withdrawing recognition from all class C colleges. It has been a cherished object of this Board to become a class A Board, and as soon as sufficient time elapses for educational conditions to adjust themselves in Texas, that all of our own institutions can be given a class A rating by the Council of Medical Education of the A. M. A., this Board will admit none but the graduates of A colleges to take our examination.

This brief history is given in order that the reader may familiarize himself with a few of the things accomplished by this Board during the seven years of its existence. Texas today classes with New York, Pennsylvania and seventeen of the leading States of the Union. In fact, our entrance requirements are higher than those of either New York or Pennsylvania, or many of the other States, for the reason that their entrance requirements are fixed by statute, while ours is left to the discretion of the Board. The medical colleges now in Texas class with the better class of medical schools in the United States. It is astonishing to contemplate such great changes as have taken place in Texas in such a short time. Those who have had the responsibility of conducting medical colleges in Texas have been called upon to accomplish in seven years what the schools of Northern and Eastern States did in a quarter of a century, and I am not far from the truth when I state that more real progress has been made in Texas in the advancement of medical education in the past seven years than has been made in any other State in the past twenty-five years. The truthfulness of this statement can be verified by a comparison of the working of this Board and of the advancements of our medical colleges with the other States of the Union. There is at this time absolutely no need of any student leaving Texas to seek a medical education.

---

Following is the list of questions propounded by the State Board of Medical Examiners at Austin, June 23d, 24th and 25th:

### PHYSIOLOGY.

W. B. COLLINS, M. D.

1. What is the physiological difference between plants and animals? 2. Give the functions of the blood as a whole, and of the red cells and leukocytes independently. 3. What are the agencies which may be brought into activity to resist the harmful effect of foreign substances in the blood stream? 4. Why does packing a wound with gauze hasten clotting? 5. Why should distilled water not be injected into the blood vessels? 6. What are the sources of the red and white blood cells? 7. What conditions of the circulatory system may lead to fainting? 8. How can we afford the greatest voluntary assistance to the entrance of venous blood into the heart? 9. Explain the occurrence of a venous pulse. 10. What different vascular conditions may lead to a paling of the face? 11. Why does putting a cold object, such as a key, down the back, tend to stop epistaxis? 12. Would the division of the cervical sympathetic nerve have any effect on the color of the face?

### HISTOLOGY.

1. What is a cell? 2. Describe a sebaceous gland. 3. Describe the preparation of a block of tissue for the microscope. 4. What is the normal histology of an ovary? 5. Define, and describe different forms of an epithelial cell. 6. Give histology of the liver. 7. Name juices of the stomach. 8. Give histology of the mammary glands. 9. Give histology of the spinal cord. 10. Describe histology of the arachnoid membrane.

### ANATOMY.

1. Describe the fourth cervical vertebra, and distinguish it from the fourth dorsal. 2. What structures would be served in an amputation at the lower third of the arm? 3. What articulations have an interarticular fibrocartilage? 4. Give brief outline of the arrangement and plan of distribution of the sympathetic nerve system. 5. Name the arteries supplying intestinal tract from pylorus to anus. 6. Give location of the thoracic duct, and indicate what portions of the body are drained by it. 7. Describe (a) the superior longitudinal sinus; (b) lateral sinus; (c) torcular herophili. 8. Name the blood vessels that constitute the portal venous system, and the organs from which each conveys the blood. 9. Give the origin, insertion, action, blood and nerve supply of the following muscles: Occipito-frontalis, pectoralis major, deltoid, adductor longus, rectus abdominus, and sterno mastoid. 10. Name all the cranial nerves, and give their functions.

### OBSTETRICS.

G. L. BABER, M. D.

1. Give the care and management of a case of gestation from beginning of third month to delivery. 2. Give all the positive signs of pregnancy, and how early can each be determined. 3. How would you manage an automatic abortion? 4. Give measurements of superior strait; the inferior strait. 5. How would you do a cephalic version? 6. Give management of third stage of labor. 7. Describe a justo-major and minor pelvis, and give etiology of same. 8. Differentiate ectopic gestation from ovarian cyst. 9. Give five complications that may arise during labor, and give management of same. 10. Give the mechanism of labor.

### PHYSICAL DIAGNOSIS.

S. L. SCOTHORN, D. O.

1. What is the significance of the patella reflex? What does it indicate when exaggerated? When absent? 2. Give normal blood pressure for adult man of 30; and state in what diseases we would most likely observe hypertension and hypotension. 3. How would you differentiate between the urethra, the bladder, and the kidneys, as the sources of pus in the urine? 4. What is the cause of the impulse felt in a scrotal hernia on coughing; when is this impulse absent in such a hernia; and in what other condition resembling hernia may it be present? 5. What is the difference between a sinus and a fistula? 6. Give the differential diagnosis of chancre, chancroid, and herpes-progenitalis. 7. What are the physical signs of stenosis of the mitral valve? 8. Make a differential diagnosis of apoplexy, opium poisoning, and alcoholic intoxication. 9. What are the differential points between conjunctivitis, iritis, and glaucoma? 10. Define volvulus, and intussusception.

### MEDICAL JURISPRUDENCE.

M. E. DANIEL, M. D.

1. It being as clearly the function of a medico-legal investigation to clear the innocent as to convict the guilty, describe (a) a post-mortem in a medico-legal case, (b) in a case of strychnine poisoning. 2. For medico-legal purposes, give the differential characteristics in the identification of human bones as applies to sex, age, race, height. 3. What is a wound; give classification; differentiate suicidal and homicidal incised wounds of the neck. 4. What is a fracture; how may fractures cause death; differentiate fractures in the living and in the dead body. 5. How would you distinguish burns inflicted before and after death; give medico-legal importance. 6. A man is found dead in a cellar; "foul play" is suspected; arrests made pending investigation; "choke damp" or "sewer gas" might be responsible for his death. As an expert, you are called in to determine cause of death. Realizing innocence or criminal conviction depends upon your report, how would you proceed—give post-mortem findings? 7. Differentiate justifiable, criminal and feigned abortion; how would you prove the latter? 8. In order to convict for infanticide, give four positive elements of criminality that the law requires. 9. The relation of insanity to the law depends upon two questions; name them; what is meant by a lucid interval, and what is the importance of its recognition? 10. Name the eight classifications of insanity.

### SURGERY.

E. B. OSBORN, M. D.

1. Describe usual deformity in fracture of anatomical neck of femur; also in surgical neck, and give treatment of each condition. 2. Differential diagnosis orchitis and epididymitis. Causation of each. 3. Plaintiff alleges fracture—dislocation between ninth dorsal and third lumbar vertebrae. Demonstrate fact that he is not malingering. Give cardinal symptoms of such injury. 4. Define sepsis, asepsis and antisepsis, germicide, parasiticide and disinfectant. 5. What is meant by surgical shock? Can pyemia produce shock? 6. Amputate forearm at lower third, giving technique in detail. 7. How does esophageal stricture produce death? How would you treat same? 8. Symptoms of irritation of phrenic nerve. Consequence of bilateral paralysis this nerve; and due to supply what parts does it produce such results? Fracture-dislocation what vertebrae would produce such paralysis? 9. Why, when and what part of body would you do lumbar puncture? 10. What artery is of interest to surgeon when he does venesection through median basilic vein? Reason for same.

### BACTERIOLOGY.

H. C. MORROW, M. D.

1. Differentiate strict or obligative bacteria and sacrophytic bacteria. 2. Name the different modes by which bacteria propagate. 3. Name the principal bacteria which may be found in the affected portions of the intestinal canal in a fatal case of typhoid fever from inception until death occurs. 4. Describe how the opsonic index is taken, and what is its value in the treatment of diseases. 5. Name at least one disease in which the following bacteria are invariably present: Streptococcus pyogenes aureus, staphylococcus pyogenes aureus, spirochetae obermeieri, gonococcus, diplococcus lanceolatus, bacillus coli communis, Klebs-Lœffler. 6. Give name, morphology and classification of at least one microbe whose habitat may be either animal, vegetable or mineral. 7. How many types of pleurisy, and the bacterial cause of each? 8. Define and discuss symbiosis, chemotaxis and phagocytosis. 9. Define the following terms: (a) coccus, (b) bacillus, (c) spirillum, (d) obligative anaerobe, (e) facultative anaerobe, (f) diplococcus, (g) mycelium, (h) sporogenous, and name at least one example of each except mycelium. 10. Name the principal culture media, and describe and give in detail the method of making a culture with any medium with which you are familiar.

### CHEMISTRY.

T. J. CROWE, M. D.

1. State the law or rule governing the diffusion of gases. 2. Name the chemical compound represented in the formula: $Na_2CO_310H_2O$. 3. What is glycerine? Give chemical formula, and a test for same. 4. Describe the chemical changes that occur in fats during digestion and assimilation. 5. Define a glucocide; name and state the source of some of the most important ones. 6. What characteristic factor—with few exceptions—distinguish the organic from the inorganic acids? Name an exception. 7. Define cataphoresis and say how it may be utilized therapeutically. 8. Name a chemical antedote for each of the following: Bichloride of mercury, nitric acid, arsenic and iodine. 9. How would you determine the urea content of a specimen of urine? 10. What would the presence of ammonia, nitrites or chlorides in a specimen of water indicate?

### GYNECOLOGY.

M. L. CROSTHWAIT, M. D.

1. Differentiate between tubal pregnancy and chronic pyosalpynx. 2. Describe two pathological conditions demanding amputation of the cervix uteri, and briefly outline the technic of the operation. 3. Define (a) subinvolution of the uterus, (b) retroversion, (c) anteversion of the uterus, and (d) prolapse of the uterus. 4. Discuss briefly the physiological functions of the ovary. 5. Describe urethral caruncle; give the diagnosis and treatment of same. 6. Mention (a) the non-malignant tumors, (b) the malignant tumors that may affect the uterus. 7. Give ten causes of intermenstrual hemorrhage in married women. 8. Differentiate between acites and ovarian cysts. 9. Mention the pathological conditions affecting the mammary gland demanding the radical removal of the breast, and what data would you require before advising the operation. 10. Mention five causes and five symptoms of chronic cystitis in women.

---

DR. W. A. HARPER, of Austin, has returned from a three weeks' visit to the eye, ear and throat hospitals of Chicago.

